# 1-{3-(4-Chloro­phen­yl)-5-[4-(propan-2-yl)phen­yl]-4,5-di­hydro-1*H*-pyrazol-1-yl}butan-1-one

**DOI:** 10.1107/S1600536814013063

**Published:** 2014-06-11

**Authors:** B. Narayana, Vinutha V. Salian, Balladka K. Sarojini, Jerry P. Jasinski

**Affiliations:** a Department of Studies in Chemistry, Mangalore University, Mangalagangotri 574 199, India; b Department of Studies in Chemistry, Industrial Chemistry Section, Mangalore University, Mangalagangotri 574 199, India; c Department of Chemistry, Keene State College, 229 Main Street, Keene, NH 03435-2001, USA

## Abstract

In the title compound, C_22_H_25_ClN_2_O, the pyrazole ring exhibits an envelope conformation with the methine C atom as the flap. The benzene rings are twisted by 3.3 (5) and 84.6 (5)° from the pyrazole mean plane, and are inclined to each other by 81.4 (4)°. In the crystal, pairs of weak C—H⋯O hydrogen bonds form centrosymmetric dimers with an *R*_2_^2^(16) graph-set motif. C—H⋯π inter­actions link the dimers into columns propagating in [100].

## Related literature   

For the biological activity of pyrazolines, see: Samshuddin *et al.* (2012*a* ▶,*b* ▶). For related structures, see: Baktır *et al.* (2011 ▶); Jasinski *et al.* (2010 ▶); Fun *et al.* (2012*a* ▶,*b* ▶); Samshuddin *et al.* (2010 ▶, 2012*c* ▶). For puckering parameters, see: Cremer & Pople (1975 ▶). For a related structure, see: Narayana *et al.*, (2014 ▶).
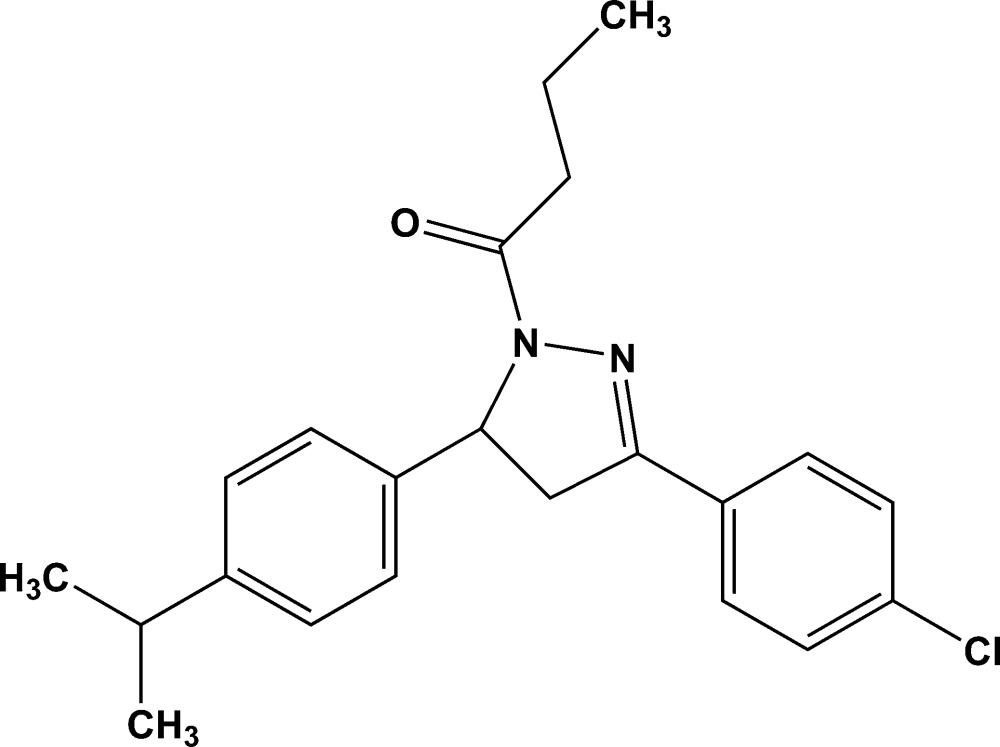


## Experimental   

### 

#### Crystal data  

C_22_H_25_ClN_2_O
*M* *_r_* = 368.89
Triclinic, 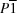

*a* = 6.8148 (6) Å
*b* = 11.1115 (9) Å
*c* = 13.8239 (15) Å
α = 70.935 (9)°
β = 81.420 (8)°
γ = 75.829 (7)°
*V* = 956.52 (17) Å^3^
*Z* = 2
Cu *K*α radiation
μ = 1.86 mm^−1^
*T* = 173 K
0.48 × 0.24 × 0.12 mm

#### Data collection  

Agilent Eos Gemini diffractometer
Absorption correction: multi-scan (*CrysAlis PRO* and *CrysAlis RED*; Agilent, 2012 ▶) *T*_min_ = 0.777, *T*_max_ = 1.000
6121 measured reflections
3624 independent reflections
3104 reflections with *I* > 2σ(*I*)
*R*_int_ = 0.028

#### Refinement  

*R*[*F* ^2^ > 2σ(*F* ^2^)] = 0.047
*wR*(*F* ^2^) = 0.134
*S* = 1.03
3624 reflections
238 parameters
H-atom parameters constrained
Δρ_max_ = 0.36 e Å^−3^
Δρ_min_ = −0.29 e Å^−3^

### 

Data collection: *CrysAlis PRO* (Agilent, 2012 ▶); cell refinement: *CrysAlis PRO*; data reduction: *CrysAlis RED* (Agilent, 2012 ▶); program(s) used to solve structure: *SUPERFLIP* (Palatinus *et al.*, 2012 ▶); program(s) used to refine structure: *SHELXL2012* (Sheldrick, 2008 ▶); molecular graphics: *OLEX2* (Dolomanov *et al.*, 2009 ▶); software used to prepare material for publication: *OLEX2*.

## Supplementary Material

Crystal structure: contains datablock(s) I. DOI: 10.1107/S1600536814013063/cv5461sup1.cif

Structure factors: contains datablock(s) I. DOI: 10.1107/S1600536814013063/cv5461Isup2.hkl

Supporting information file. DOI: 10.1107/S1600536814013063/cv5461Isup3.cml

CCDC reference: 1006822

Additional supporting information:  crystallographic information; 3D view; checkCIF report

## Figures and Tables

**Table 1:** Hydrogen-bond geometry (Å, °) *Cg* is the centroid of the C5–C10 ring.

*D*—H⋯*A*	*D*—H	H⋯*A*	*D*⋯*A*	*D*—H⋯*A*
C15—H15⋯O1^i^	0.95	2.57	3.437 (2)	151
C20—H20*A*⋯*Cg* ^ii^	0.99	2.67	3.5079 (19)	143

Symmetry codes: (i) 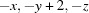; (ii) 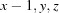.

**Fig. 1.**
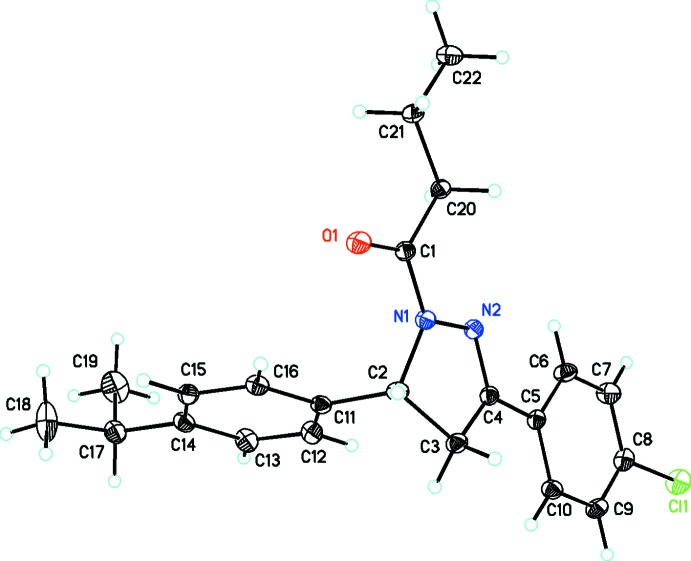
The molecular structure of (I) showing the labeling scheme and 30% probability displacement ellipsoids.

**Fig. 2.**
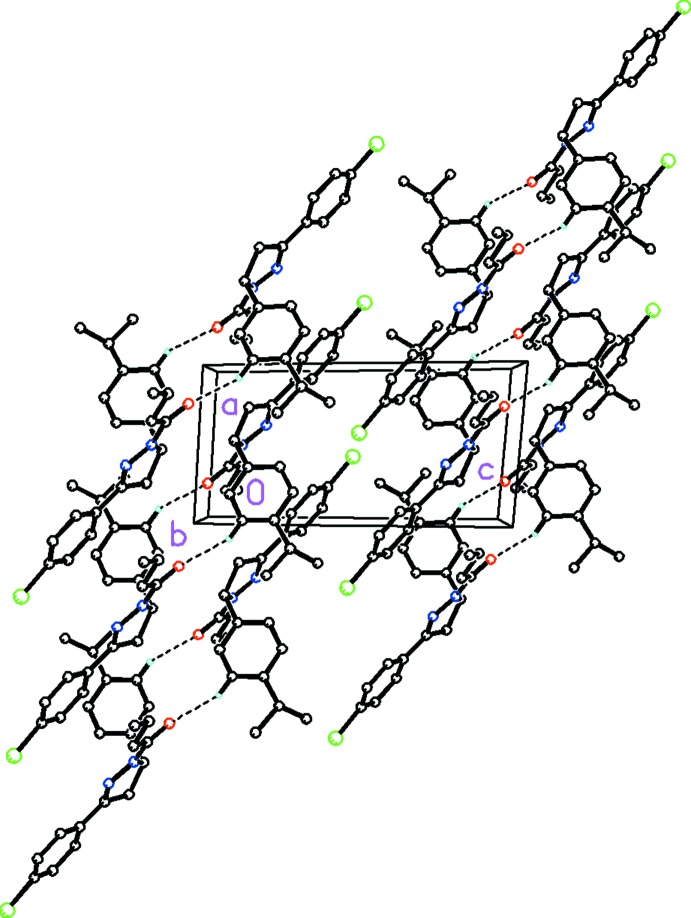
Molecular packing for (I) viewed along the <b axis. Dashed lines indicate weak C—H···O hydrogen bonds. H atoms not involved with these weak intermolecular interactions have been removed for clarity.

**Fig. 3.**
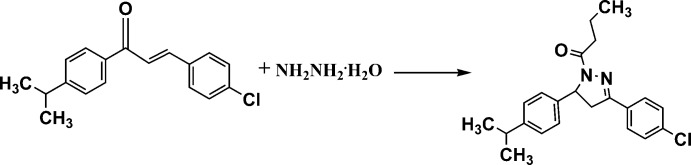
Synthesis of (I).

## supplementary crystallographic information

### S1. Comment

Pyrazoline derivatives are biologically active compounds. They possess activities like antimicrobial, analgesic and antioxidant activities (Samshuddin *et al.*, 2012*a*,*b*). The crystal structure of some pyrazoline derivatives viz., 3,5-bis(4-fluorophenyl)-4,5-dihydro-1H-pyrazole-1-carbaldehyde (Baktır *et al.*, 2011), 3,5-bis(4-fluorophenyl)-1-phenyl-4,5-dihydro-1H-pyrazole (Jasinski *et al.*, 2010), 5-(4-bromophenyl)-3-(4-fluorophenyl)-1-phenyl-4,5-dihydro-1H-pyrazole, 1-[5-(4-bromophenyl)-3-(4-fluorophenyl)-4,5-dihydro-1H-pyrazol-1-yl] butan-1-one (Fun *et al.*, 2012*a*,*b*) and 3,5-bis(4-bromophenyl)-1-phenyl-4,5-dihydro-1H-pyrazole, 3,5-bis(4-fluorophenyl)-1-(4-nitrophenyl)-4,5-dihydro-1H-pyrazole (Samshuddin *et al.*, 2010, 2012*c*) have been reported. Herein we report the crystal structure of the title compound (I).

In (I) (Fig. 1), the pyrazole ring exhibits an envelope conformation (puckering parameters Q =0.1957 (19)Å, φ = 314.1 (5)°; Cremer & Pople, 1975) with the methine carbon atom as a flap. Bond lengths are in normal ranges and correspond to those observed in the related structures (Baktır *et al.*, 2011; Jasinski *et al.*, 2010; Fun *et al.*, 2012*a*,*b*; Samshuddin *et al.*, 2010, 2012*c*). The two benzene rings are twisted at 3.3 (5)° and 84.6 (5)°, respectively, from the pyrazole mean plane, and are inclined to each other at 81.4 (4)°.

In the crystal, a weak C—H···O intermolecular interaction between the phenyl ring and the butan-1-one group is observed forming dimers in an R_2_^2^[16] ring-set motif (Fig. 2). In addition, weak C—H···π intermolecular stacking interactions (Table 1) are also present and link further these dimers into columns propagated in [100].

### S2. Experimental

To a mixture of (2E)-1-(4-chlorophenyl)-3-[4-(propan-2-yl) phenyl] prop-2-en-1-one (2.85 g, 0.01 mol) and hydrazine hydrate (0.5 mL, 0.01 mol) in 30 mL butyric acid was refluxed for 10h (Fig. 3). The reaction mixture was cooled and poured into 50 mL ice-cold water. The precipitate formed was collected by filtration and purified by recrystallization from ethanol. Single crystals were grown from DMF by the slow evaporation method (m.p.: 369–371 K).

### S3. Refinement

All H atoms were placed in their calculated positions and refined using the riding model with C—H of 0.95 - 1.00 Å. Isotropic displacement parameters for H atoms were set to 1.2–1.5 times *U*_eq_ of the parent atom. The idealised Me was refined as a rotating group.

### Crystal data

**Table:**

C_22_H_25_ClN_2_O	*Z* = 2
*M**_r_* = 368.89	*F*(000) = 392
Triclinic, *P*1	*D*_x_ = 1.281 Mg m^−^^3^
*a* = 6.8148 (6) Å	Cu *K*α radiation, λ = 1.54184 Å
*b* = 11.1115 (9) Å	Cell parameters from 2535 reflections
*c* = 13.8239 (15) Å	θ = 4.6–71.4°
α = 70.935 (9)°	µ = 1.86 mm^−^^1^
β = 81.420 (8)°	*T* = 173 K
γ = 75.829 (7)°	Prism, colourless
*V* = 956.52 (17) Å^3^	0.48 × 0.24 × 0.12 mm

### Data collection

**Table:**

Agilent Eos Gemini diffractometer	3624 independent reflections
Radiation source: Enhance (Cu) X-ray Source	3104 reflections with *I* > 2σ(*I*)
Graphite monochromator	*R*_int_ = 0.028
Detector resolution: 16.0416 pixels mm^-1^	θ_max_ = 71.3°, θ_min_ = 3.4°
ω scans	*h* = −8→8
Absorption correction: multi-scan (*CrysAlis PRO* and *CrysAlis RED*; Agilent, 2012)	*k* = −13→10
*T*_min_ = 0.777, *T*_max_ = 1.000	*l* = −16→16
6121 measured reflections	

### Refinement

**Table:**

Refinement on *F*^2^	Primary atom site location: structure-invariant direct methods
Least-squares matrix: full	Hydrogen site location: inferred from neighbouring sites
*R*[*F*^2^ > 2σ(*F*^2^)] = 0.047	H-atom parameters constrained
*wR*(*F*^2^) = 0.134	*w* = 1/[σ^2^(*F*_o_^2^) + (0.0787*P*)^2^ + 0.1856*P*] where *P* = (*F*_o_^2^ + 2*F*_c_^2^)/3
*S* = 1.03	(Δ/σ)_max_ < 0.001
3624 reflections	Δρ_max_ = 0.36 e Å^−^^3^
238 parameters	Δρ_min_ = −0.29 e Å^−^^3^
0 restraints	

### Special details

**Table:**

Geometry. All esds (except the esd in the dihedral angle between two l.s. planes) are estimated using the full covariance matrix. The cell esds are taken into account individually in the estimation of esds in distances, angles and torsion angles; correlations between esds in cell parameters are only used when they are defined by crystal symmetry. An approximate (isotropic) treatment of cell esds is used for estimating esds involving l.s. planes.

### Fractional atomic coordinates and isotropic or equivalent isotropic displacement parameters (Å^2^)

**Table:**

	*x*	*y*	*z*	*U*_iso_*/*U*_eq_	
Cl1	1.42326 (7)	0.34338 (5)	0.49295 (4)	0.04081 (18)	
O1	0.25099 (19)	0.72366 (12)	0.02504 (10)	0.0295 (3)	
N1	0.4990 (2)	0.67625 (14)	0.12995 (11)	0.0225 (3)	
N2	0.6268 (2)	0.58597 (14)	0.20187 (11)	0.0213 (3)	
C1	0.3613 (2)	0.64081 (16)	0.08821 (13)	0.0216 (3)	
C2	0.5284 (3)	0.81112 (16)	0.10752 (13)	0.0229 (4)	
H2	0.5346	0.8553	0.0318	0.027*	
C3	0.7387 (3)	0.78265 (17)	0.14768 (13)	0.0249 (4)	
H3A	0.8467	0.7902	0.0909	0.030*	
H3B	0.7440	0.8420	0.1868	0.030*	
C4	0.7574 (2)	0.64368 (16)	0.21642 (13)	0.0214 (3)	
C5	0.9147 (2)	0.57270 (17)	0.28833 (12)	0.0218 (3)	
C6	0.9295 (3)	0.43990 (18)	0.33925 (15)	0.0297 (4)	
H6	0.8325	0.3970	0.3298	0.036*	
C7	1.0828 (3)	0.37023 (19)	0.40300 (15)	0.0337 (4)	
H7	1.0922	0.2799	0.4370	0.040*	
C8	1.2233 (3)	0.43392 (19)	0.41678 (13)	0.0276 (4)	
C9	1.2096 (3)	0.56538 (19)	0.37002 (14)	0.0285 (4)	
H9	1.3048	0.6081	0.3816	0.034*	
C10	1.0544 (3)	0.63514 (18)	0.30554 (14)	0.0258 (4)	
H10	1.0437	0.7259	0.2731	0.031*	
C11	0.3582 (3)	0.88801 (16)	0.16102 (13)	0.0221 (3)	
C12	0.3619 (3)	0.88196 (18)	0.26294 (14)	0.0285 (4)	
H12	0.4768	0.8312	0.2994	0.034*	
C13	0.2016 (3)	0.94830 (18)	0.31206 (14)	0.0306 (4)	
H13	0.2081	0.9421	0.3817	0.037*	
C14	0.0295 (3)	1.02465 (17)	0.26082 (14)	0.0259 (4)	
C15	0.0259 (3)	1.03183 (16)	0.15890 (14)	0.0254 (4)	
H15	−0.0885	1.0833	0.1222	0.030*	
C16	0.1878 (3)	0.96452 (16)	0.10962 (13)	0.0237 (4)	
H16	0.1820	0.9709	0.0398	0.028*	
C17	−0.1436 (3)	1.09614 (19)	0.31842 (15)	0.0314 (4)	
H17	−0.0822	1.1473	0.3488	0.038*	
C18	−0.3049 (4)	1.1923 (3)	0.25140 (19)	0.0543 (7)	
H18A	−0.2410	1.2535	0.1947	0.081*	
H18B	−0.4025	1.2404	0.2924	0.081*	
H18C	−0.3755	1.1453	0.2237	0.081*	
C19	−0.2392 (4)	1.0005 (3)	0.40755 (19)	0.0535 (6)	
H19A	−0.2975	0.9461	0.3812	0.080*	
H19B	−0.3464	1.0487	0.4447	0.080*	
H19C	−0.1352	0.9450	0.4542	0.080*	
C20	0.3585 (2)	0.49736 (16)	0.12252 (13)	0.0222 (4)	
H20A	0.3351	0.4671	0.1982	0.027*	
H20B	0.4928	0.4484	0.1035	0.027*	
C21	0.1959 (3)	0.46751 (17)	0.07518 (13)	0.0245 (4)	
H21A	0.2187	0.4978	−0.0005	0.029*	
H21B	0.0612	0.5156	0.0945	0.029*	
C22	0.1971 (3)	0.3223 (2)	0.11055 (16)	0.0347 (4)	
H22A	0.0947	0.3061	0.0762	0.052*	
H22B	0.1660	0.2932	0.1850	0.052*	
H22C	0.3314	0.2742	0.0931	0.052*	

### Atomic displacement parameters (Å^2^)

**Table:**

	*U*^11^	*U*^22^	*U*^33^	*U*^12^	*U*^13^	*U*^23^
Cl1	0.0383 (3)	0.0454 (3)	0.0359 (3)	−0.0011 (2)	−0.0233 (2)	−0.0053 (2)
O1	0.0329 (7)	0.0260 (6)	0.0298 (7)	−0.0028 (5)	−0.0177 (5)	−0.0045 (5)
N1	0.0239 (7)	0.0197 (7)	0.0240 (7)	−0.0018 (6)	−0.0114 (6)	−0.0046 (6)
N2	0.0219 (7)	0.0210 (7)	0.0206 (7)	−0.0006 (5)	−0.0085 (5)	−0.0055 (5)
C1	0.0216 (8)	0.0246 (9)	0.0204 (8)	−0.0033 (6)	−0.0054 (6)	−0.0086 (6)
C2	0.0263 (8)	0.0212 (8)	0.0216 (8)	−0.0063 (7)	−0.0085 (6)	−0.0032 (6)
C3	0.0231 (8)	0.0243 (9)	0.0276 (9)	−0.0054 (7)	−0.0070 (7)	−0.0058 (7)
C4	0.0203 (8)	0.0237 (8)	0.0211 (8)	−0.0036 (6)	−0.0035 (6)	−0.0082 (6)
C5	0.0201 (8)	0.0269 (9)	0.0192 (8)	−0.0026 (6)	−0.0046 (6)	−0.0084 (7)
C6	0.0297 (9)	0.0288 (9)	0.0325 (10)	−0.0099 (7)	−0.0110 (7)	−0.0054 (8)
C7	0.0385 (10)	0.0272 (10)	0.0329 (10)	−0.0058 (8)	−0.0148 (8)	−0.0012 (8)
C8	0.0260 (9)	0.0373 (10)	0.0187 (8)	−0.0017 (7)	−0.0096 (7)	−0.0077 (7)
C9	0.0262 (9)	0.0375 (10)	0.0265 (9)	−0.0091 (7)	−0.0086 (7)	−0.0113 (8)
C10	0.0278 (9)	0.0257 (9)	0.0262 (9)	−0.0056 (7)	−0.0071 (7)	−0.0086 (7)
C11	0.0251 (8)	0.0171 (8)	0.0242 (8)	−0.0050 (6)	−0.0075 (6)	−0.0035 (6)
C12	0.0322 (9)	0.0251 (9)	0.0261 (9)	0.0016 (7)	−0.0163 (7)	−0.0042 (7)
C13	0.0390 (10)	0.0309 (10)	0.0228 (9)	−0.0016 (8)	−0.0118 (7)	−0.0092 (7)
C14	0.0318 (9)	0.0199 (8)	0.0258 (9)	−0.0051 (7)	−0.0058 (7)	−0.0055 (7)
C15	0.0271 (9)	0.0199 (8)	0.0281 (9)	−0.0027 (6)	−0.0116 (7)	−0.0033 (7)
C16	0.0309 (9)	0.0214 (8)	0.0193 (8)	−0.0058 (7)	−0.0098 (7)	−0.0030 (6)
C17	0.0348 (10)	0.0280 (9)	0.0312 (10)	−0.0023 (8)	−0.0064 (8)	−0.0101 (8)
C18	0.0466 (13)	0.0582 (15)	0.0393 (12)	0.0204 (11)	−0.0043 (10)	−0.0122 (11)
C19	0.0593 (15)	0.0477 (14)	0.0426 (13)	−0.0076 (11)	0.0094 (11)	−0.0075 (11)
C20	0.0224 (8)	0.0237 (9)	0.0221 (8)	−0.0034 (6)	−0.0069 (6)	−0.0078 (7)
C21	0.0241 (8)	0.0279 (9)	0.0251 (9)	−0.0063 (7)	−0.0055 (7)	−0.0108 (7)
C22	0.0393 (11)	0.0331 (10)	0.0386 (11)	−0.0137 (8)	−0.0063 (8)	−0.0141 (8)

### Geometric parameters (Å, º)

**Table:**

Cl1—C8	1.7443 (17)	C12—C13	1.382 (3)
O1—C1	1.228 (2)	C13—H13	0.9500
N1—N2	1.3903 (18)	C13—C14	1.402 (2)
N1—C1	1.363 (2)	C14—C15	1.388 (2)
N1—C2	1.486 (2)	C14—C17	1.527 (2)
N2—C4	1.289 (2)	C15—H15	0.9500
C1—C20	1.511 (2)	C15—C16	1.395 (3)
C2—H2	1.0000	C16—H16	0.9500
C2—C3	1.537 (2)	C17—H17	1.0000
C2—C11	1.515 (2)	C17—C18	1.513 (3)
C3—H3A	0.9900	C17—C19	1.522 (3)
C3—H3B	0.9900	C18—H18A	0.9800
C3—C4	1.512 (2)	C18—H18B	0.9800
C4—C5	1.467 (2)	C18—H18C	0.9800
C5—C6	1.397 (3)	C19—H19A	0.9800
C5—C10	1.394 (2)	C19—H19B	0.9800
C6—H6	0.9500	C19—H19C	0.9800
C6—C7	1.378 (3)	C20—H20A	0.9900
C7—H7	0.9500	C20—H20B	0.9900
C7—C8	1.388 (3)	C20—C21	1.518 (2)
C8—C9	1.376 (3)	C21—H21A	0.9900
C9—H9	0.9500	C21—H21B	0.9900
C9—C10	1.394 (2)	C21—C22	1.524 (3)
C10—H10	0.9500	C22—H22A	0.9800
C11—C12	1.392 (2)	C22—H22B	0.9800
C11—C16	1.394 (2)	C22—H22C	0.9800
C12—H12	0.9500		
			
N2—N1—C2	112.45 (13)	C12—C13—C14	121.12 (17)
C1—N1—N2	121.93 (14)	C14—C13—H13	119.4
C1—N1—C2	125.61 (14)	C13—C14—C17	119.15 (16)
C4—N2—N1	107.90 (14)	C15—C14—C13	117.76 (16)
O1—C1—N1	119.87 (16)	C15—C14—C17	123.09 (16)
O1—C1—C20	123.77 (15)	C14—C15—H15	119.5
N1—C1—C20	116.35 (14)	C14—C15—C16	120.95 (16)
N1—C2—H2	110.2	C16—C15—H15	119.5
N1—C2—C3	100.23 (13)	C11—C16—C15	121.13 (16)
N1—C2—C11	110.45 (14)	C11—C16—H16	119.4
C3—C2—H2	110.2	C15—C16—H16	119.4
C11—C2—H2	110.2	C14—C17—H17	106.8
C11—C2—C3	115.22 (14)	C18—C17—C14	114.41 (17)
C2—C3—H3A	111.4	C18—C17—H17	106.8
C2—C3—H3B	111.4	C18—C17—C19	110.51 (19)
H3A—C3—H3B	109.2	C19—C17—C14	111.04 (16)
C4—C3—C2	101.99 (13)	C19—C17—H17	106.8
C4—C3—H3A	111.4	C17—C18—H18A	109.5
C4—C3—H3B	111.4	C17—C18—H18B	109.5
N2—C4—C3	113.43 (14)	C17—C18—H18C	109.5
N2—C4—C5	120.71 (15)	H18A—C18—H18B	109.5
C5—C4—C3	125.71 (15)	H18A—C18—H18C	109.5
C6—C5—C4	120.24 (15)	H18B—C18—H18C	109.5
C10—C5—C4	120.94 (16)	C17—C19—H19A	109.5
C10—C5—C6	118.80 (16)	C17—C19—H19B	109.5
C5—C6—H6	119.5	C17—C19—H19C	109.5
C7—C6—C5	120.99 (17)	H19A—C19—H19B	109.5
C7—C6—H6	119.5	H19A—C19—H19C	109.5
C6—C7—H7	120.5	H19B—C19—H19C	109.5
C6—C7—C8	119.10 (17)	C1—C20—H20A	109.0
C8—C7—H7	120.5	C1—C20—H20B	109.0
C7—C8—Cl1	118.71 (15)	C1—C20—C21	112.72 (14)
C9—C8—Cl1	119.96 (14)	H20A—C20—H20B	107.8
C9—C8—C7	121.32 (16)	C21—C20—H20A	109.0
C8—C9—H9	120.4	C21—C20—H20B	109.0
C8—C9—C10	119.24 (17)	C20—C21—H21A	109.3
C10—C9—H9	120.4	C20—C21—H21B	109.3
C5—C10—H10	119.8	C20—C21—C22	111.64 (15)
C9—C10—C5	120.49 (17)	H21A—C21—H21B	108.0
C9—C10—H10	119.8	C22—C21—H21A	109.3
C12—C11—C2	121.95 (15)	C22—C21—H21B	109.3
C12—C11—C16	117.72 (16)	C21—C22—H22A	109.5
C16—C11—C2	120.30 (15)	C21—C22—H22B	109.5
C11—C12—H12	119.3	C21—C22—H22C	109.5
C13—C12—C11	121.31 (16)	H22A—C22—H22B	109.5
C13—C12—H12	119.3	H22A—C22—H22C	109.5
C12—C13—H13	119.4	H22B—C22—H22C	109.5
			
Cl1—C8—C9—C10	177.00 (14)	C3—C2—C11—C16	153.55 (15)
O1—C1—C20—C21	−3.4 (2)	C3—C4—C5—C6	−172.73 (17)
N1—N2—C4—C3	−3.92 (19)	C3—C4—C5—C10	5.9 (3)
N1—N2—C4—C5	−179.60 (13)	C4—C5—C6—C7	176.61 (17)
N1—C1—C20—C21	177.93 (14)	C4—C5—C10—C9	−176.72 (15)
N1—C2—C3—C4	−18.43 (16)	C5—C6—C7—C8	0.3 (3)
N1—C2—C11—C12	84.01 (19)	C6—C5—C10—C9	1.9 (3)
N1—C2—C11—C16	−93.79 (18)	C6—C7—C8—Cl1	−177.13 (15)
N2—N1—C1—O1	179.87 (15)	C6—C7—C8—C9	1.5 (3)
N2—N1—C1—C20	−1.4 (2)	C7—C8—C9—C10	−1.6 (3)
N2—N1—C2—C3	18.47 (17)	C8—C9—C10—C5	−0.1 (3)
N2—N1—C2—C11	−103.49 (15)	C10—C5—C6—C7	−2.0 (3)
N2—C4—C5—C6	2.4 (2)	C11—C2—C3—C4	100.09 (16)
N2—C4—C5—C10	−179.00 (15)	C11—C12—C13—C14	−0.3 (3)
C1—N1—N2—C4	171.21 (15)	C12—C11—C16—C15	−0.5 (2)
C1—N1—C2—C3	−162.79 (16)	C12—C13—C14—C15	−0.3 (3)
C1—N1—C2—C11	75.2 (2)	C12—C13—C14—C17	−179.98 (18)
C1—C20—C21—C22	179.69 (15)	C13—C14—C15—C16	0.4 (3)
C2—N1—N2—C4	−10.00 (19)	C13—C14—C17—C18	170.5 (2)
C2—N1—C1—O1	1.3 (3)	C13—C14—C17—C19	−63.6 (2)
C2—N1—C1—C20	180.00 (15)	C14—C15—C16—C11	0.0 (3)
C2—C3—C4—N2	15.17 (19)	C15—C14—C17—C18	−9.2 (3)
C2—C3—C4—C5	−169.40 (15)	C15—C14—C17—C19	116.7 (2)
C2—C11—C12—C13	−177.21 (17)	C16—C11—C12—C13	0.6 (3)
C2—C11—C16—C15	177.37 (15)	C17—C14—C15—C16	−179.92 (16)
C3—C2—C11—C12	−28.6 (2)		

### Hydrogen-bond geometry (Å, º)

Cg is the centroid of the C5–C10 ring.

**Table:**

*D*—H···*A*	*D*—H	H···*A*	*D*···*A*	*D*—H···*A*
C15—H15···O1^i^	0.95	2.57	3.437 (2)	151
C20—H20*A*···*Cg*^ii^	0.99	2.67	3.5079 (19)	143

Symmetry codes: (i) −*x*, −*y*+2, −*z*; (ii) *x*−1, *y*, *z*.
